# Long non-coding RNA HULC as a potential prognostic biomarker in human cancers: a meta-analysis

**DOI:** 10.18632/oncotarget.15247

**Published:** 2017-02-10

**Authors:** Yang-Hua Fan, Miao-Jing Wu, Yuan Jiang, Minhua Ye, Shi-Gang Lu, Lei Wu, Xin-Gen Zhu

**Affiliations:** ^1^ Department of Neurosurgery, The Second Affiliated Hospital of Nanchang University, Nanchang 330006, Jiangxi Province, People's Republic of China

**Keywords:** HULC, neoplasm, metastasis, prognosis, meta-analysis

## Abstract

Since the long non-coding RNA HULC (Highly Upregulated in Liver Cancer) is dysregulated in many cancers, we performed a meta-analysis to determine its prognostic potential in malignant tumors. We searched electronic databases, including PubMed, Medline, OVID, Cochrane Library and Web of Science from inception until August 14, 2016 and identified seven studies with 730 cancer patients for the meta-analysis. We analyzed the hazard ratios (HRs) and 95% confidence intervals (CIs) to determine the relationship between HULC expression and overall survival (OS). We also using RevMan5.3 software to calculate odds ratio (ORs) to assess the association between HULC expression and pathological parameters, including lymph node metastasis (LNM), distant metastasis (DM) and the tumor stage. Our analysis showed that higher HULC expression was associated with OS (HR= 0.50, 95% CI: 0.35–0.70, P <0.00001), LNM (OR=0.20, 95 % CI 0.06–0.64), DM (OR=0.27, 95% CI: 0.13–0.54) and the tumor stage (OR=0.39, 95 % CI 0.25–0.64). These meta-analysis data demonstrate that higher HULC expression can be a useful prognostic biomarker in human cancers.

## INTRODUCTION

In 2012, nearly 8.2 million people reportedly died from cancer and about 14.1 million people were diagnosed with cancer worldwide [1]. The American National Center for Health Statistics has estimated that nearly 600 thousand Americans will die of cancer in 2016 [2]. The five year survival rate of most cancers is extremely low and since survival depends on early diagnosis of cancer, there is a constant need to identify and develop newer diagnostic and prognostic markers.

Long noncoding RNA (lncRNA) are transcribed RNA molecules that lack an open reading frame and are longer than 200 nucleotides [3]. They are involved in epigenetic regulation, transcriptional and posttranscriptional regulation that are key cellular processes that also determine tumorigenesis [4]. Dysregulation of lncRNAs has been reported in many types of cancers [5–8]. Since they have been implicated in different stages of cancer progression including proliferation, invasion and metastasis, they are promising prognostic markers for cancer [9–10, 11]. Due to the specific expression of lncRNA in the development of tumor and their presence in body fluids and tumor tissues, they are promising biomarkers to diagnose and monitor tumors [12]. Therefore, identification of tumor related lncRNAs that are vital in tumorigenesis are promising biomarkers for cancer prognosis.

HULC (Highly Upregulated in Liver Cancer) was first reported in liver cancer and showed extensive regulatory functions in cell proliferation, apoptosis, invasion, cell cycle, and drug resistance [13]. HULC is an lncRNA with two non-translated exons and about 500 nucleotides long. It is highly expressed in hepatocellular carcinoma and colorectal cancer with liver metastasis [14]. Since HULC expression has been shown during cancer growth and metastasis, it is a promising prognostic biomarker candidate for human cancers [15]. In recent years, HULC has been found to be dysregulated in osteosarcoma, pancreatic cancer, colorectal cancer, hepatocellular carcinoma, gastric cancer, hepatocellular carcinoma and large B-cell lymphoma [16–22]. Du and others found that the HULC promoted liver cancer cell proliferation by inhibiting P18 [23]. Silencing of HULC effectively reversed EMT phenotype in gastric cancer [24]. These studies revealed that HULC has potential prognostic value in cancer patients. However, most studies regarding HULC are limited by discrete outcomes and small patient samples. Therefore, we performed this meta-analysis to determine the prognostic value of HULC by combined analysis of data from multiple studies.

## RESULTS

### Literature search analysis results

**Figure 1:**
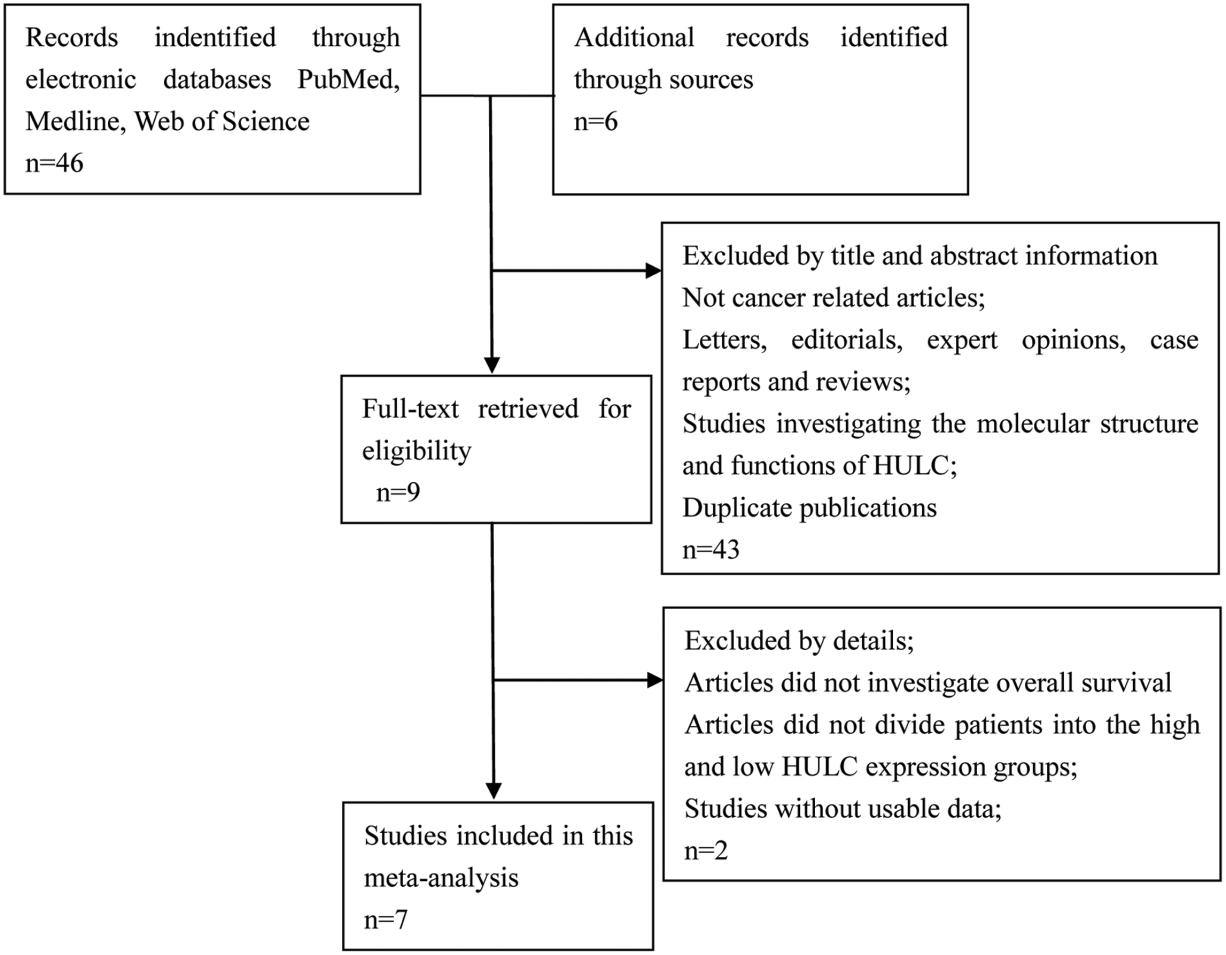
Flowchart showing the steps of literature search and selection criteria for the meta-analysis

The detailed screening process of HULC studies is shown in Figure 1. Based on the inclusion and exclusion criteria, a total of seven studies and 730 patients were included in the meta-analysis [16–22]. The characteristics of the seven studies are summarized in Table 1. The total number of subjects analyzed in the seven studies ranged from 33 to 304, with a mean sample size of 104.3. Six of the seven studies were conducted in China whereas one study was from Brazil and were published between 2014 and 2016. Among the seven studies, two focused on osteosarcoma (OSC) [20–21] and one each on gastric cancer [16], hepatocellular carcinoma [17], pancreatic cancer [18], large B-cell lymphoma [19] and colorectal cancer [22]. HULC expression was measured in the tumor specimen as well as the serum. The diagnosis of LNM, DM and tumor stage depended on the pathology. The NOS scores of all the studies were ≥7.

**Table 1:** The basic information and data of all included studies in the meta-analysis

Study	Year	Region	Tumor type	Age	Men %	Reference gene	Sample size	HULC expression						Analysis (OS)	HR(95% CI)Low/High	NOS	Method
								Low			High						
								Total	LNM	DM	Total	LNM	DM				
Jin^[16]^	2016	China	GC	60	65	GAPDH	100	52	25	2	48	34	9	Multivariate	0.58(0.23-1.44)	8	qRT-PCR
Li^[17]^	2016	China	HCC	-	76.3	GAPDH	38	15	-	1	23	-	8	Multivariate	0.47(0.11-2.01)	8	qRT-PCR
Peng^[18]^	2014	China	PC	-	55.3	GAPDH	304	92	23	-	212	157	-	Multivariate	0.352(0.172-0.752)	7	qRT-PCR
Peng^[19]^	2016	China	LBCL	-	70.4	GAPDH	142	47	-	-	95	-	-	Multivariate	0.738(0.414-1.288)	7	qRT-PCR
Sun^[20]^	2015	China	OSC	-	57.7	GAPDH	78	39	-	5	39	-	16	Multivariate	0.439(0.184-0.675)	8	qRT-PCR
Uzan^[21]^	2016	Brazil	OSC	5	48.5	GAPDH	33	21	-	-	12	-	-	Multivariate	0.045(0.0046–0.443)	7	qRT-PCR
Yang^[22]^	2016	China	CRC	-	-	GAPDH	35	12	-	-	23	-	-	Multivariate	0.43 (0.05-4.04)	8	qRT-PCR

Note: The dashes represent no data.

Abbreviations: GAPDH, glyceraldehyde-3-phosphate dehydrogenase; GC, gastric cancer; HCC, hepatocellular carcinoma; PC, pancreatic cancer; LBCL, large B-cell lymphoma; OSC, osteosarcoma; CRC, colorectal cancer; LNM, lymph node metastasis; DM, distant metastasis.

### Association between HULC expression levels and OS

**Figure 2:**
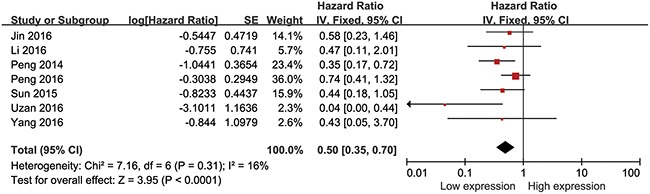
Forest plot showing association between OS and elevated HULC expression in the different types of cancer

We performed cumulative meta-analysis to determine the role of HULC in overall survival (OS) of all 730 cancer patients from the seven studies. Statistical analyses revealed that HULC was associated with OS of cancer patients (pooled HR= 0.50, 95% CI: 0.35–0.70, P <0.00001; Figure 2). Our analyses did not find any significant heterogeneity among the studies (I^2^=16%, P_Q_=0.31). Therefore, our data demonstrated that HULC was an independent OS factor among cancer patients and its high expression was associated with shorter OS.

### Association between HULC expression level and LNM

**Figure 3:**
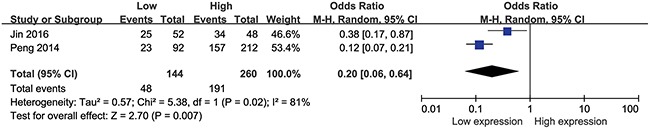
Forest plot showing association between HULC expression levels and lymph node metastasis

Data of 404 cancer patients from 2 eligible studies was collected and analyzed. The random effects model was used due to significant heterogeneity (I^2^=81%, P=0.02) and the odds ratio (OR) determined as 0.20 (95% CI: 0.06–0.64, P=0.007; Figure 3). Although our analysis demonstrated significant differences in the LNM incidence between the two groups, patients with higher expression of HULC were more prone to developing LNM.

### Association between HULC expression levels and DM

**Figure 4:**
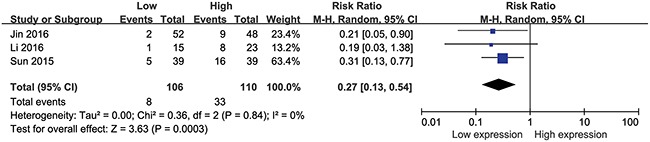
Forest plot showing association between HULC expression levels and distant metastasis

Analysis of 216 patients from three eligible studies demonstrated association between HULC expression levels and the number of cancer patients with DM (Figure 4). Analysis by the fixed effects model showed no significant heterogeneity (I^2^=0%, P_Q_=0.84) and the pooled OR was 0.27 (95% CI: 0.13–0.54, P=0.0003; Figure 4). These results indicated that patients with high HULC expression level in the tumor tissues may have increased probability of DM.

### Association between HULC expression levels and tumor stage

**Figure 5:**
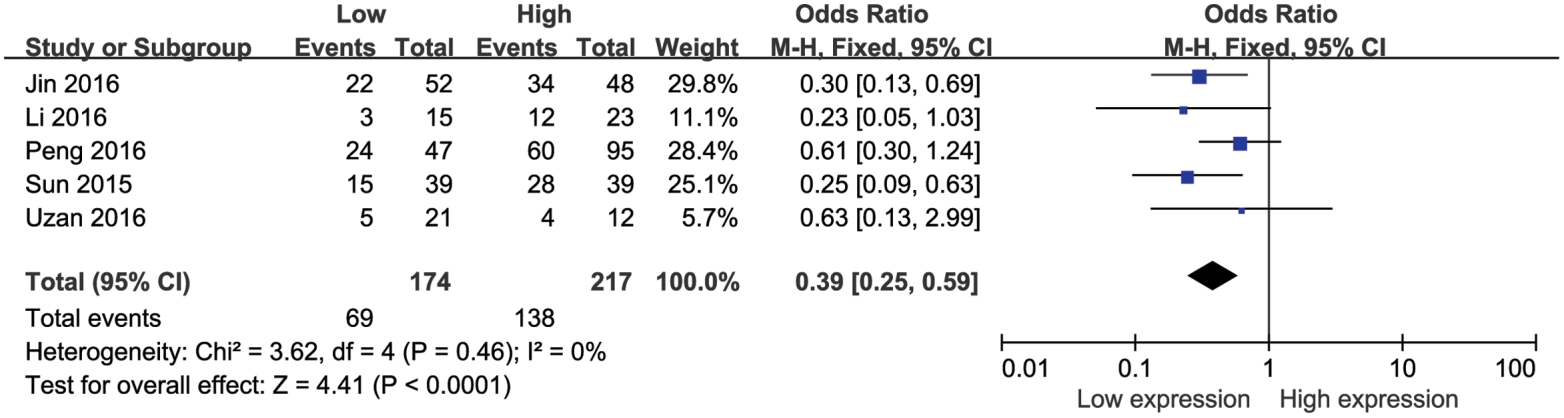
Forest plot showing meta-analysis of the role of HULC on tumor stage in the different types of cancer

A total of 391 patients from five eligible studies were analyzed for the relationship between the HULC expression levels and the tumor stage in this meta-analysis. Our data demonstrated that higher the HULC expression, higher was the tumor grade with a pooled OR of 0.39 (95% CI: 0.25–0.59, P <0.0001; Figure 5) with no obvious heterogeneity (I^2^=16%, P_Q_=0.31). Therefore, our results demonstrated that higher expression of HULC significantly increased the risk of high tumor stage.

### Begg's funnel plot analysis

**Figure 6:**
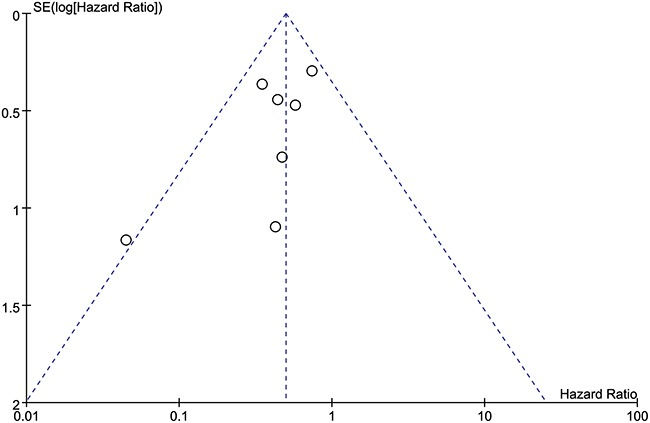
Funnel plot analysis to determine publication bias for the independent role of HULC on OS in the different types of cancers

**Figure 7:**
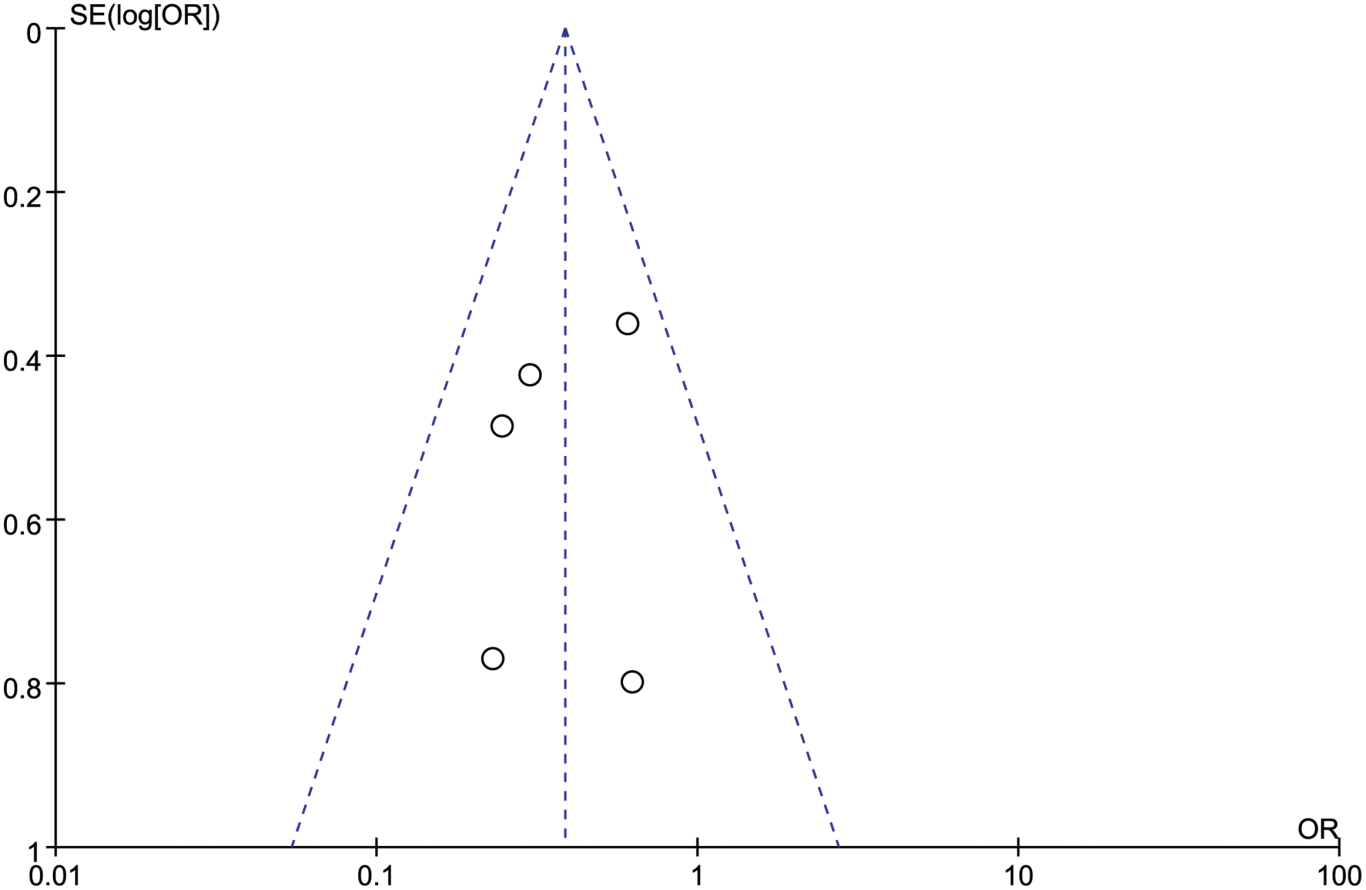
Funnel plot analysis to determine publication bias for the independent role of HULC on tumor stage in the different types of cancers

We did not find any obvious asymmetry for either overall survival (Figure 6) or tumor stage (Figure 7) when we used Begg's funnel plot to analyze publication bias. Therefore, our findings were due to a relationship between HULC expression and the pathological parameters analyzed and not due to publication bias.

## DISCUSSION

Cancer is a major threat to human health all over the world and the incidence of cancer has increased gradually over the years [2]. Most cancers eventually metastasize as lymph node metastasis (LNM) and distant metastasis (DM). The occurrence of metastasis indicates poor prognosis and hence is an important indicator for survival. [25–26]. Moreover, LNM and DM are important for the diagnosis of TNM (tumor–node–metastasis) staging for cancer patients, as well as important indicators for predicting prognosis. Since the precise mechanism on metastasis remains unknown in most cancers, molecular biomarkers play a critical role in the diagnosis, prognosis and treatment of cancer [27–28]. Therefore finding new molecular markers that accurately predict tumor metastasis are of paramount importance.

Since the role of HULC as a molecular biomarker in human cancer was unclear, our study explored the prognostic value of HULC in cancer patients using the meta-analysis.

A random effects model or fixed effects model was used to analyze the data based on the results of heterogeneity analysis. Our data showed that higher HULC expression was indicative of advanced cancer and highlighted poor prognosis. By combining HRs from Cox multivariate analyses, there was a significant difference in OS between high and low HULC expression level groups (pooled HR 0.50, 95%CI: 0.35–0.70). In addition, we showed that higher HULC expression was associated with lymph node metastasis, distant metastasis and tumor stage. We found that high HULC expression was significantly associated with advanced tumor stages without obvious heterogeneity in different types of cancer. Due to lack of literature we could not perform meta-analysis to find if higher HULC expression in tumor issues may be related to the recurrence time [17], progression–free survival (PFS) [19] and event-free survival (EFS) [21]. We also found that HULC is a promising marker for diagnosis in tumor [24]. In gastric cancer, a receiver operating characteristic (ROC) curve was constructed to assess the diagnostic utility of serum HULC and the area under the ROC curve was determined to be 0.888, which was higher than the well known tumor markers for gastrointestinal malignancies, CA72-4 and CEA [16]. In addition, Peng and others found that the area under the ROC curve was 0.977 in pancreatic cancer [18] and 0.9765 in large B-cell lymphoma [19]. Therefore, HULC can be considered an independent diagnostic marker in cancer patients.

Nevertheless, there are few limitations that must be taken into account while interpreting the conclusions of our meta-analysis. First, most studies were from China, and only one study was from Brazil. Therefore our data may not represent globally. Second, the included type and number of cancers were small. Third, the criterion for high expression varied for different studies. Therefore, further well-designed and high-quality studies are needed to confirm the function of HULC in various cancers.

In conclusion, since high levels of HULC expression in multiple cancers are associated with poor OS, LNM, DM and tumor stage, it is a promising prognostic biomarker in cancer.

## MATERIALS AND METHODS

### Literature search to identify relevant studies for meta-analysis

A systematic search of multiple electronic databases, Medline, Pubmed, OVID, and Web of Science, was performed independently by two authors, Yanghua Fan and Lei Wu in accordance with the standard guidelines of meta-analysis. They searched literature from inception until August 14, 2016 for articles that reported HULC as a probable prognostic marker for survival of cancer patients [29–30]. The searches were performed by both the text word and MeSH strategy and included terms like ‘HULC’, ‘Highly upregulated in liver cancer’, ‘hepatocellular carcinoma up-regulated long non-coding RNA’, ‘lncRNA’, ‘noncoding RNA’,‘long intergenic noncoding RNA’, ‘carcinoma’, ‘neoplasm’, ‘tumor’, ‘cancer’, ‘prognostic’, ‘prognosis’, ‘outcome’, ‘survival’ or ‘recurrence’. The strategy was adjusted in different databases to maximize finding the appropriate articles. Manual searches were also performed using the reference lists of the relevant articles to retrieve eligible studies for inclusion in meta-analysis.

### Selection criteria for including studies in meta-analysis

The two researchers, Yanghua Fan and Minhua Ye, independently evaluated all the data in the articles to select relevant studies for meta-analysis. The criteria used to include studies in the meta-analysis were as follows: 1) The relationship between HULC expression and survival was measured in multiple human tumors; 2) The expression level of HULC was measured either in human tumor tissue or serum and the patients were grouped according to the expression levels of HULC; 3) All tumors were confirmed by pathological or histological examinations and the pathological parameters like LNM, DNM, tumor stage were described; 4) Studies described statistical information on overall survival (OS) such as hazard ratio (HR) and 95% confidence interval (CI).

The criteria to exclude studies from the meta-analysis included: 1) The articles that were reviews, letters, editorials, case reports and expert opinions; 2) Non-English language and non-human studies; 3) Studies lacking data listed in the criteria for included studies; 4) Basic characterization studies of HULC.

### Data extraction from relevant studies for meta-analysis

The two reviewers, Yanghua Fan and Lei Wu, independently extracted and examined the data from the selected original articles. Any disagreements in the literature assessment were resolved through consensus with a third reviewer, Xingen Zhu. The following details were collected from each of the study: surname of the first author, publication year, country, tumor type, median age of patients, percentage of male, reference gene, sample size, the number of patients with lymph node metastasis and distant metastasis, HR and 95% CI of elevated HULC for OS, the Newcastle-Ottawa Scale (NOS) score and the detection method of HULC.

The study quality was assessed in accordance with the Newcastle-Ottawa Scale (NOS). A total of nine items, each of which was assigned a score of 1, were measured in each study. The total scores for different studies ranged from 0 to 9. If the score was ≥7, the study was considered to be of high quality.

### Statistical analysis

The statistical analysis was performed by RevMan version 5.3 software. The heterogeneity among different studies was measured by the Q and I^2^ tests. A probability value of I^2^ ≥50% and P < 0.1 indicated the existence of significant heterogeneity [31]. A random effects model or fixed effects model was selected based on the results of heterogeneity analysis. The random-effects model was used if there was significant heterogeneity among the studies or else, the fixed effects model was used. The potential publication bias was assessed by the Begg's funnel plot. Pooled HRs and ORs were obtained from the published data. We used the HRs and 95% CIs reported in a publication when it was available and when they were not reported, the HR values were estimated from the survival information obtained from Kaplan-Meier curve. Overall survival was calculated using the log HR and standard error (SE) values [32]. Odds ratios (ORs) and their 95 % CIs were used to assess the association between HULC expression and the tumor parameters, including LNM, DM and tumor stage.
